# (Re) Solving Repair After Myocardial Infarction

**DOI:** 10.3389/fphar.2018.01342

**Published:** 2018-11-26

**Authors:** Giovanna Leoni, Oliver Soehnlein

**Affiliations:** ^1^Institute for Cardiovascular Prevention (IPEK), University of Munich, Munich, Germany; ^2^German Center for Cardiovascular Research (DZHK), Partner Site Munich Heart Alliance, Munich, Germany; ^3^Department of Physiology and Pharmacology (FyFa), Karolinska Institute, Stockholm, Sweden; ^4^Department of Medicine, Karolinska Institute, Stockholm, Sweden

**Keywords:** cardiac, ischemia, inflammation, resolution, repair

## Abstract

Cardiovascular diseases, including myocardial infarction and its complications such as heart failure, are the leading cause of death worldwide. To date, basic and translational research becomes necessary to unravel the mechanisms of cardiac repair post-myocardial infarction. The local inflammatory tissue response after acute myocardial infarction determines the subsequent healing process. The diversity of leukocytes such as neutrophils, macrophages and lymphocytes contribute to the clearance of dead cells while activating reparative pathways necessary for myocardial healing. Cardiomyocyte death triggers wall thinning, ventricular dilatation, and fibrosis that can cause left ventricular dysfunction and heart failure. The ultimate goal of cardiac repair is to regenerate functionally viable myocardium after myocardial infarction to prevent cardiac death. Current therapies for heart failure after myocardial infarction are limited and non-curative. At the moment in clinic, conventional surgical interventions such as coronary artery bypass graft or percutaneous coronary interventions are only able to partially restore heart function, with a minor improvement in the left ventricular ejection fraction. The goal of this review is to provide an overview of endogenous myocardial repair mechanisms possibly transferable to future treatment strategies. Among the innovative factors identified as essential in cardiac healing, we highlight specialized pro-resolving mediators as the emerging factors that provide the key molecular signals for the activation of the reparative cells in the myocardium.

## Introduction

**FIGURE 1:**
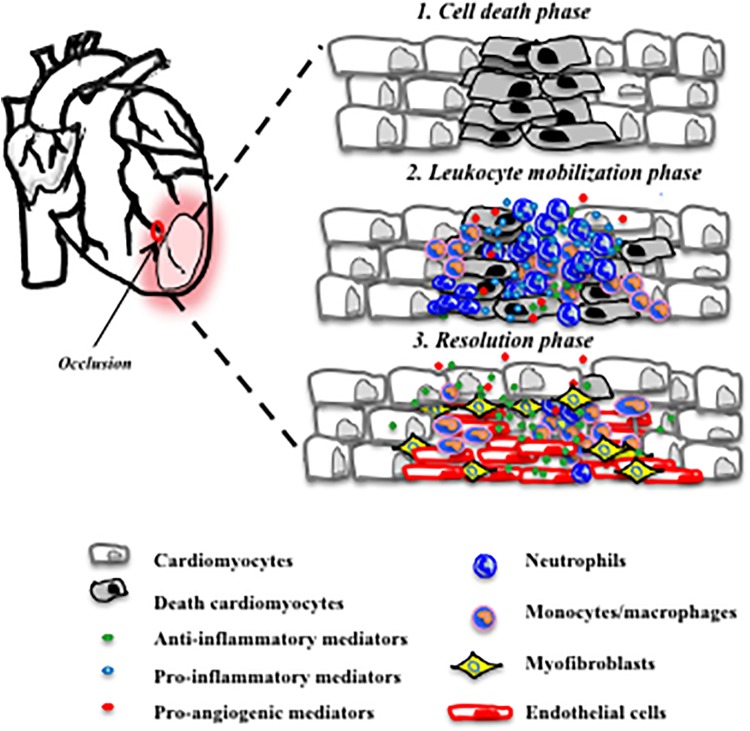
Three major phases post-myocardial infarction. Cardiomyocyte death (Benjamin et al., 2018) recruitment of neutrophils and pro-inflammatory monocytes (Frangogiannis, 2012) release of anti-inflammatory mediators and promotion of angiogenesis and repair (Swirski et al., 2009).

Cardiovascular such as myocardial infarction, diseases, are the leading cause of morbidity and mortality worldwide, causing 31% of all global deaths (Benjamin et al., 2018). A history of acute myocardial infarction is associated with a 5-fold increase in the incidence of heart failure after 5 years of myocardial infarction. Therefore, there is a need to prevent cardiac failure by enhancing cardiac repair processes. Following infarction, the myocardium undergoes major changes both in its function and structure (Frangogiannis, 2012). Immediately after myocardial infarction, a robust inflammatory reaction occurs: immune cells mainly, neutrophils and monocytes, migrate into the heart, due to the release of myocardial danger-associated molecular patterns (DAMPs) derived by necrotic and stressed/injured cardiac cells (cardiomyocytes). Later, the resolution phase lasts a few days to weeks and encompasses the reparative or resolving phase. Finally, the progression phase lasts months or years depending on the resolution phase, which, if defective, leads to cardiac dysfunction, chronic heart failure, and mortality (Frangogiannis, 2012). A representative image shows the different stages of myocardial infarction (Figure 1). In the post-myocardial infarction initiation phase, numerous leukocytes travel from the splenic reservoir through the circulation to the myocardium that generates an edematous inflammatory milieu (Swirski et al., 2009). DAMPs bind to cognate pattern recognition receptors of the innate immune system on infiltrating leukocytes and activate the release of inflammatory cytokines, chemokines and activate cell adhesion molecules. The initial recruitment of immune cells can promote cardiac fibrosis and heart failure (Epelman et al., 2015). However, only recently studies show that immune cells also contribute to the repair process and the acute inflammatory response is more recently seen and described as essential and protective. Several studies in fact, report that controlling inflamed leukocytes promote cardioprotection (Nahrendorf et al., 2015). The balance between inflammation and resolution becomes crucial for the cardiac functionality, inappropriate inflammation delays the myocardial repair process. At the moment, improvement of intrinsic wound healing has emerged as a potential strategy to prevent heart failure.

## Cardiac Repair Post-Myocardial Infarction Is a Superbly Orchestrated Process

During myocardial infarction, neutrophil infiltration occurs immediately peaking at day 1 (Hansen, 1995). Neutrophils are pivotal players in post-infarction healing by potentially favoring the recruitment of inflammatory monocytes (Nahrendorf et al., 2007; Soehnlein et al., 2008). These cells present at the surface chemokine receptors (CCR) such as CCR2. CCR2 expression changes in a time-of-day–dependent manner, which crucially affects cardiac monocyte recruitment during myocardial infarction (Schloss et al., 2017). Experimental evidence also suggests that neutrophils directly damage cardiomyocytes in the myocardium through the release of toxic products, such as high amount of reactive oxygen species (Vinten-Johansen, 2004). However, in addition, data also demonstrate that neutrophils can improve cardiac function and cardiac repair (Horckmans et al., 2017). Recent studies indicate that neutrophils may acquire different phenotypes and contribute to resolution of inflammation through the release of anti-inflammatory mediators. Thus, neutrophils have been proposed to shift toward, pro-resolving/N2 phenotype instead of a N1 pro-inflammatory phenotype to promote tissue repair in condition of myocardial infarction (Ma et al., 2016). Neutrophils have both beneficial and detrimental roles during myocardial infarction, depending on their phenotype: too many N1 neutrophils damage tissue and cells leading to more inflammation. Too few N2 neutrophils may not be able to promote resolution of inflammation and apoptotic cardiomyocyte clearance: the perfect balance of N1 and N2 neutrophils becomes necessary for optimal cardiac repair. Achieving this balance represents the ideal pro-resolving conditions for patients with myocardial infarction (Romson et al., 1983; Ma et al., 2013, 2016; Carbone et al., 2016; Horckmans et al., 2017).

Macrophages represent another abundant cell population after myocardial infarction. They remain predominant in the infarcted left ventricle during the late phases of myocardial infarction (Yan et al., 2013; Hilgendorf et al., 2014). Macrophages regulate multiple aspects of the cardiac healing response, such as clearance of dead cells *via* Tyrosine-protein kinase Mer activation during myocardial infarction (DeBerge et al., 2017). Macrophages are classified in inflammatory macrophages (M1) during the initial phase of myocardial infarction and anti-inflammatory macrophages (M2) in the later phase of myocardial infarction (Nahrendorf et al., 2007; Troidl et al., 2009). M1 macrophages display the classical M1 surface marker expressing Ly-6C^high^ and CD206^low^ and higher levels of pro-inflammatory mediators (nitric oxide synthase, IL-6 IL-1b, and IL-12a). M2 macrophages express Ly-6C^low^ and CD206^high^ with pro-resolving signature genes such as IL-10, arginase-1, and TGF-b. Interestingly, M2 macrophages mediate the beneficial effects of bone marrow-derived mesenchymal stromal cells in infarct healing and repair (Ben-Mordechai et al., 2013).

Among all the cells that contribute to the cardiac functionally there are also lymphocytes, observed in patients that had myocardial infarction (Nunez et al., 2008). Lymphocytes, consisting of T cells, B cells, and natural killer (NK) cells have important roles in both innate and adaptive immune responses in myocardial infarction. However, not much attention has been paid to these cells in the context of cardiac healing. Regulatory cells also often have potent effects, despite their relative scarcity (Epelman and Mann, 2012). Proliferative T cells: Th cells (CD4), cytotoxic T cells (CD8), and Foxp3 + regulatory CD4 + T cells are present in heart draining lymph nodes (Hofmann et al., 2012). During myocardial infarction, T cells number increases, due to the recruitment in the heart, since there are no studies reporting any increase of lymphocyte proliferation. B- and T-cell levels reach the peak after 7 days of myocardial infarction (Yan et al., 2013). Studies reported that patients with myocardial infarction have lower CD4+ but higher CD8+ T lymphocytes (Blum and Yeganeh, 2003; Liu et al., 2011; Yan et al., 2015). CD4+ T lymphocytes can differentiate into Th1 and Th2 lineage in response to the local milieu of cytokines during myocardial infarction. Th2 cells show protective role during myocardial infarction (Engelbertsen et al., 2013). NK cells are cytotoxic lymphocytes critical to the acute immune system during myocardial infarction (Yan et al., 2015). Not much is known about B lymphocytes during myocardial infarction. However, several studies using for example, mice deficient in B cells, demonstrate their crucial role during ischemia/reperfusion models (Kalogeris et al., 2012; Zouggari et al., 2013).

The inflammatory response that occurs during myocardial infarction is seen as an important element for the clearance of dead cells and the stimulation of the reparative processes. If dying cells are not eliminated this can further promote permanent loss of cardiac functionality and heart failure. The process of cardiac repair involves phagocytosis/clearance of apoptotic cells in the heart, predominantly promoted by macrophages, but other non-professional phagocytes have been shown to participate in this process such as cardiomyocytes and fibroblasts. Fibroblasts during myocardial infarction become activated and differentiate into myofibroblasts (Dutta et al., 2015; Nakaya et al., 2017). Cardiomyocytes can phagocytose latex particles *in vitro* (Garfield et al., 1975) and potentially cardiomyocyte debris *in vivo* (Hurle et al., 1977, 1978). Myofibroblasts mediated clearance of dying cells after myocardial infarction *via* milk fat globule epidermal growth factor (Han et al., 2016). Myofibroblasts are capable of other roles, such as extracellular matrix metabolism, contractile activity, producing and secreting greater levels of extracellular matrix proteins, including several types of collagen, important to strengthen the infarct and to protect it against rupture and neovessel formation (Frangogiannis et al., 2002). During myocardial infarction injury, cardiac fibroblasts interact with cardiomyocytes and this interaction is important for the heart to heal and recover (Fu et al., 2018). Other interactions among extracellular matrix, endothelial cells, and macrophages are also important for cardiac repair and neovessel formation/angiogenesis (Carmeliet, 2000). Angiogenic agents such as vascular endothelial growth factor (VEGF) and basic fibroblast growth factor (bFGF) are rapidly released in the ischemic myocardium and facilitate growth of blood flow vessels, heart tissue repair and prevent the onset of heart failure (Zhao et al., 2010).

## Resolving but Not Dampening Inflammation for Cardiac Healing

Maintaining the optimal balance of inflammation is crucial to induce myocardial healing (Kain et al., 2014). Several experimental studies have shown a better outcome in infarcted myocardium using anti-inflammatory treatment. However, some of the anti-inflammatory treatments failed in clinical practice (Silverman and Pfeifer, 1987; Saxena et al., 2016). As consequence, current guidelines recommend against the use of broad-range anti-inflammatory therapy corticosteroids and non-steroidal anti-inflammatory drugs–in patients with acute myocardial infarction (Task Force on the management of St-segment elevation acute myocardial infarction of the European Society of Cardiology (ESC) et al., 2012). In fact, the inhibition of COX-2 and TNF-α reduce cardiac functionality post-infarct in patients and COX-2 inhibitors (rofecoxib and celecoxib) in clinical settings accelerate the myocardial infarction events (Chung et al., 2003; Saito et al., 2003; Mann et al., 2004; Kimmel et al., 2005; Antman et al., 2007; Skyschally et al., 2007; Listing et al., 2008; Kleinbongard et al., 2010). Also a recombinant IL-1 receptor antagonist increase rates of recurrent myocardial infarction within 12 months, however, a larger study evaluating longer term IL-1 inhibition is active at the moment (Ridker et al., 2011; Morton et al., 2015). Possible reasons for failure of anti-inflammatory agents for myocardial infarction in clinical trials are that preclinical studies mostly involve healthy and young animals, unlike human patients (with different ages and gender) that often present chronic comorbidities. Another possible reason is that the targets were often non-specific. Diagnostic techniques such as tomography may be used to develop goal directed therapies (Herrero et al., 2007).

Promotion of cardiac repair is a key therapeutic goal against the failure of survival during ischemia. Low oxygen content that occurs during myocardial infarction is the major cause of this cardiac cell death. Re-introducing oxygen into the infarcted area represents a promising goal. A novel oxygen delivery system able to continuously release oxygen to the infarct area, protects the cardiac cells, could represent a new therapeutic approach for patients with myocardial infarction. The system was based on a thermosensitive injectable and fast gelation hydrogel, characterized by its capacity of oxygen releasing microspheres. With this technique the release of oxygen lasts 4 weeks and it significantly increases survival of cardiac cells, neovessel formation, and cardiac functionality, under the hypoxic condition that mimicked the infarcted hearts (Fan et al., 2018). Treatments with angiogenic or anti-apoptotic factors are very promising for improving cardiac functionality in condition of myocardial infarction. Despite the use of pro-angiogenic factors in clinical trials has led to good results in the improvement of cardiac function, there are still difficulties to overcome. In fact, clinical trials involving VEGF or bFGF do not have the expected beneficial effects (Hedman et al., 2003; Henry et al., 2003; Kukula et al., 2011). For example, a proper spatio-temporal delivery of multiple therapeutic proteins represents a major challenge in therapy strategies aimed to induce myocardial regeneration after myocardial infarction and on the other hand the pro-angiogenic growth factors expression has to be very tightly regulated in order to avoid side effects such as the promotion of tumor growth (Alfranca, 2009). Another important issue in therapeutic angiogenesis is that the delivery of a single growth factor might be insufficient to mimic the complex regulatory mechanisms driving neovascularization. Many of the strategies failed in the clinical setting and they rely upon using a single-targeted approach, directed to only one specific molecule or intracellular signaling pathway. Therefore, a multi-targeted approach directed to more than one intracellular signaling pathways may have more cardioprotective effects, considering also the presence of a co-morbidity in patients (Tsang et al., 2005). Several new approaches are discovered, such as VEGF enriched nanoparticles administration *via* local injection into the peri-infarct region is able to increase the angiogenic and therapeutic efficiency of VEGF in promoting cardiac repair (Oduk et al., 2018). VEGF-loaded microsphere patch for local protein delivery to the ischemic heart after myocardial injury in rats are even more promising: VEGF-patched hearts have better blood vessels growth, tissue repair and heart function (Rodness et al., 2016). The use of three-dimensional matrices despite the encouraging results in terms of cardiac regeneration and performance remains a relatively invasive method since they are surgically implanted over the infarct region. Drug administration in conditions of myocardial infarction at the moment includes oral or needle-based routes which can lead to patient discomfort (Suarez et al., 2015). Engineered cardiac patches are currently considered as a promising therapeutical approach for regeneration of the heart, however, their integration within the myocardium by sutures may cause further damage. A new suture-free technology for the attachment of engineered tissues positioned on the myocardium and irradiated with a laser represents an even better therapeutic approach at the moment (Malki et al., 2018). Also, inhaled calcium phosphate nanoparticles can deliver to the myocardium therapeutic compounds in a less invasive and better way (Miragoli et al., 2018). Inhalation therapy could represent a promising alternative to increase blood flow in the setting of chronic ischemia to preserve cardiac function.

Stem cells secrete high amounts of paracrine factors that can stimulate endogenous repair mechanisms (Henning, 2018). Human embryonic stem cell-derived cardiomyocytes repair the macaque monkey hearts by reducing scar tissue and improving cardiac functionality (Liu et al., 2018). Mesenchymal stem cells are at the moment under clinical investigation as a treatment for patients with advanced heart failure after myocardial infarction to improve myocardial function (Makkar et al., 2012; Malliaras et al., 2014). Injection in the myocardium of swine of human mesenchymal cell-derived extracellular vesicles (EVs) increase blood flow to ischemic myocardial tissue by stimulating capillary and arteriolar growth *via* activation of the protein kinase B/endothelial nitric oxide synthase and mitogen-activated protein kinase signaling pathways (Ponikowski et al., 2014). EVs significantly improve cardiac output and stroke volume (Potz et al., 2018). EVs containing anti-inflammatory proteins (e.g., Annexin A1) are also shown to activate wound repair circuits in another organ therefore they could also be beneficial for cardiac healing post-ischemia (Leoni et al., 2015). Annexin A1 during the acute phase of myocardial infarction present protective effects by controlling haematopoietic stem cell mobilization and inflammation (D’Amico et al., 2000; Qin et al., 2013; Qin et al., 2017; Ritchie et al., 2005). More studies are needed to explore its role during later cardiac repair events. In the context of myocardial infarction, members of EVs called exosomes are important for the regenerative effects in the myocardium. Cardiosphere-derived cell exosomes deliver in the myocardium decrease scarring and improve ejection fraction in a porcine myocardial infarction model (Gallet et al., 2017). Their beneficial effects have been demonstrated in multiple animal models and also in a phase 1 human study (Johnston et al., 2009; Makkar et al., 2012; Malliaras et al., 2012, 2014). To reverse injury post-myocardial infarction, cardiosphere-derived cells are currently in phase 2 clinical trials with scar reduction as the major endpoint.

Novel treatments to resolve inflammation during myocardial infarction become necessary. Several interesting studies demonstrate the protective role of pro-resolving mediators during the resolution phase of inflammation (Keyes et al., 2010; Kain et al., 2015; Halade et al., 2016). Well-known drugs such as statins lower permeability and reduce the transit of unfavorable inflammatory leukocytes into the infarcted tissue, consequently improving left ventricular outcome (Bauersachs et al., 2001; Ramasubbu et al., 2008; Leenders et al., 2018). Statin treatment also improve endothelial barrier function during myocardial healing in ApoE^−/−^ mice (Leenders et al., 2018). A phase III clinical trial demonstrates that a statin called rosuvastatin has beneficial effects in patients with heart failure (McMurray et al., 2009). Aspirin also, contributes to the stimulation of the generation of pro-resolving mediators (SPMs) classified as lipoxins, resolvins, protectins, maresins, and Annexin A1 (Serhan et al., 2002; Gilroy, 2005; Serhan et al., 2011; Dalli et al., 2015a; Perretti et al., 2015). Experimental models of self-resolving inflammation demonstrate their potent anti-inflammatory and pro-resolving properties in several models (Serhan, 2010). Recently, a study shows that mice treated with 15-epi-lipoxin A_4_ present improved ejection fraction after 5 days of myocardial infarction (Kain et al., 2017). Furthermore, resolvin D1 has similar cardioprotective effects (Kain et al., 2015). Two recent studies present a quantification of SPMs in the infarcted left ventricles and spleens, after myocardial infarction (Tourki and Halade, 2017; Halade et al., 2018b). Interestingly, the peak of neutrophils after 24 h post-myocardial infarction correlates with an increase of resolvin D series in the infarcted myocardium. Later, at day 5 post-myocardial infarction, N2 neutrophils represent the most amount population in the left ventricle and spleen. Interestingly, resolvin D1 activated its receptor (lipoxin A4 receptor/formyl peptide receptor-2) to promote clearance in the infarcted heart (Kain et al., 2015). Furthermore, resolvin D1 accelerate clearance of leukocytes from an infarcted area by the activation of the miRNA circuit (Halade et al., 2018a). Human artery segments and primary cultured human vascular cells generate D-series resolvins and maresins when the relevant fatty acid precursors are present, and in the absence of leukocytes (Chatterjee et al., 2017). Recently, novel molecules termed maresin conjugates in tissue regeneration (MCTR), protectin conjugates in tissue regeneration (PCTR), and resolvin conjugates in tissue regeneration (RCTR) are identified as other pro-repair inducers (Dalli et al., 2014, 2015b; Dalli and Serhan, 2018). These new compounds could represent new therapeutical treatments for patients with myocardial infarction. The discovery of lipid mediators will serve as a novel therapeutical approach based on endogenous mechanisms for treating inflammatory response through the stimulation of resolution instead of inhibiting the inflammation.

Another important factor that controls the severity of myocardial infarction is aging. Cardiac aging is a process characterized by increased levels of reactive oxygen species, genomic DNA damage and telomere and epigenetic modifications. Aging also disregulates the level of arachidonic acid post-myocardial infarction and lipoxins release, responsible for neutrophils infiltration inhibition (Takano et al., 1998; Serhan et al., 2000; Halade et al., 2016; Serhan and Levy, 2018). Aging has effects on the innate immune response, through dysregulation of pro-inflammatory cytokines such as IL-6, IL-1β, TNF-α, and TGFβ, which lead to chronic inflammation, and thus contribute to the “inflammaging phenotype,” often observed in the elderly people (Ershler and Keller, 2000; Franceschi et al., 2000; Bruunsgaard et al., 2003). In the elderly, defects in dying-cell clearance could lead to a non-resolving inflammation and maladaptive cardiac repair, thereby accelerating heart failure (Chen and Frangogiannis, 2010). Since little is known about the pro-resolving mediators in aging itself more studies are needed to assess whether in human patients pro-resolving molecules are less abundant or less effective with increasing age and how these factors impact cardiac repair. The capacity of the heart to heal after a myocardial infarction is not enough to restore normal cardiac function. Resolution of inflammation can be influenced by diet. Only a good balance of omega-3 and -6 fatty acids demonstrates protective effects on cardiovascular system (Ramsden et al., 2013). High amount of omega-6 decrease specialized pro-resolving mediators (D and E-series), also increase macrophage accumulation in the myocardium and promote cardiorenal inflammation (Halade et al., 2016). Of note, omega-3 fatty acids are known for their cardiovascular benefit or to reduce elevated triglycerides using higher doses (Smith et al., 2006). Thus, omega-3 fatty acids has positive effects on controlling inflammation, including the reduction of cytokines, endothelial cell activation and platelet aggregation, heart rate and cardiac function. A clinical phase III trial demonstrates in fact that long-term administration of omega-3 result in a significant reduction in both all-cause mortality and cardiovascular readmissions in patients with heart failure (Yancy et al., 2013). Lifestyle-related post-myocardial infarction setting opens a new future perspective studies to prevent the progression of heart failure.

**FIGURE 2:**
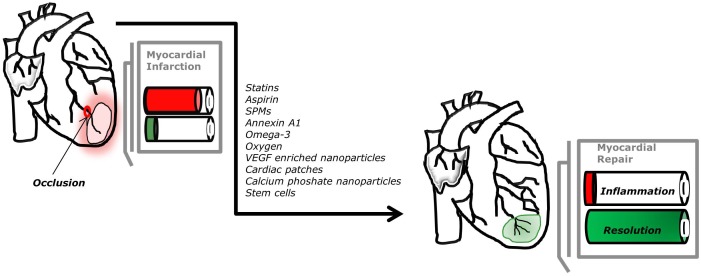
Potential new therapeutic approaches to promote myocardial repair. The optimal process of repair after myocardial infarction, arising from occlusion of the coronary circulation, requires timely induction and resolution of inflammation.

Clearly, there is still much unknown in the field of cardiac healing, nevertheless progress has been made, opening exciting new potential therapeutic options for patients affected by myocardial infarction, as shown in Figure 2. Several studies enhance the crucial role of endogenous pro-resolving mediators during myocardial infarction. Significant increases in resolvins, protectin, and maresin are observed after 1 and 5 days post-myocardial infarction and their increase correlate with leukocyte recruitment (Howlett et al., 2016). Therefore, the abundance of SPMs could also predict the risk of future cardiovascular events (Emami et al., 2015). The advantage of using pro-resolving mediators is that they act on specific cellular receptors to regulate leukocyte trafficking and blunt the release of inflammatory mediators, while also promoting clearance of dead cells and tissue repair. These mediators could inform the development of therapeutic strategies encompassing a novel resolution pharmacology approach.

In future, nanocarriers engineered to recognize pro-resolving specific receptors at the cellular levels and to deliver pro-resolving mediators into the diseased sites to subpopulations of immune cells represents a highly appealing approach to specifically improve cardiac repair.

## Author Contributions

All authors listed have made a substantial, direct, and intellectual contribution to the work, and approved it for publication.

## Conflict of Interest Statement

The authors declare that the research was conducted in the absence of any commercial or financial relationships that could be construed as a potential conflict of interest.
